# Editorial: Biorefinery chemicals: trend, sources and metrics

**DOI:** 10.3389/fchem.2023.1268526

**Published:** 2023-08-04

**Authors:** Daniele Cespi, Rafael Luque, Christophe Len, Raffaele Cucciniello

**Affiliations:** ^1^ Department of Industrial Chemistry “Toso Montanari”, University of Bologna, Bologna, Italy; ^2^ Center for Chemical Catalysis—C3, Alma Mater Studiorum Università di Bologna, Bologna, Italy; ^3^ Research Directorship, Universidad ECOTEC, Samborondón, Ecuador; ^4^ PSL Research University, Chimie ParisTech, The French National Centre for Scientific Research, Paris, France; ^5^ Department of Chemistry and Biology “Adolfo Zambelli”, University of Salerno, Fisciano, SA, Italy; ^6^ Centro Interdisciplinare Linceo Giovani, Accademia Nazionale dei Lincei Roma, Italy

**Keywords:** green chemistry, biomasses valorisation, non dedicated crops, circular economy strategies, wastes to value added products, green metrics

## Introduction

In line with the scope of promoting a Green and Sustainable Chemistry, there is the necessity to collect and share innovation on new products, techniques, sources and evaluation strategies of the greenness grade associated with products from biorefinery. Material depletion and environmental impacts implied the adoption of biomass as renewable sources for bulk and pharmaceutical chemicals. The 7th principle of green chemistry encourages the switch from a fossil to a bio-based industry to reduce burdens and minimize the waste generation. This sector of bio-based chemicals is considered among the most investigated research fields and has changed during the last two decades. Recent ambition of developing a climate neutral society seems in line with the possibility of using non-dedicated vegetable biomass to absorb carbon dioxide and stock it, at least until entering the end-of-life stage.

This Research Topic on *Biorefinery chemicals: trend, sources and metrics* covers promising and novel advances in this field by addressing new challenges within the most consolidated and used bio-based products worldwide.

## The content of this Research Topic

In this Research Topic, several applications in the field of sustainable valorization of biomasses are discussed. Therefore, we would like to thank all the authors who contributed with their excellent works to this Research Topic.

Segatto et al. investigated the usage Ionic Liquids (ILs) in the extraction of bioactive compounds from fruit waste, comparing the results with those achievable with more consolidated organic solvents like alcohols (e.g., ethanol and methanol). Firstly, they proposed the screening and optimization of aqueous solutions of ILs to obtain mangiferin and hyperoside (quercetin-3-Ogalactoside) enriched extracts from the Mango Processing Waste (MPW). In this study, commonly used ILs such as 1-alkyl-3-methyl-imidazolium cations ([CnMIm]) were compared to greener ILs (e.g., choline acetate and 2-hydroxyethylammonion formate), by selecting Homogenizer-Assisted Extraction (HAE) as quick, efficient and inexpensive extraction technique on MPW derived from commercial processing unit in Itirapina (São Paulo, Brazil). Ethyl (C2), butyl (C4), hexyl (C6), octyl (C8) and decyl (C10) variants of [CnMIm] were tested, with bromine as their anion pair, as well as 1-octyl-3-methylimidazolium chloride, the same C8 variant, but with chloride as the anion. The other selected ILs were: 1-butyl-1-methylpyrrolidinium bromide ([C_4_MPyrr] Br), choline acetate, 2-hydroxyethylammonium formate and 1-ethylpyridinium bromide. The extractions were performed using aqueous solutions of 1 M of each ionic liquid in triplicate, following the abovementioned procedure. Imidazolium-based ILs showed higher extraction recoveries for both analytes, which peaked at the 1-octyl-imidazolium cation, when compared to the other alkyl chain lengths. Comparison between two different anions paired to [C_8_MIm] showed no statistical difference between the results for mangiferin (497.0 and 520.8 mg kg^−1^ for chlorine and bromine, respectively) but a superior yield of hyperoside extraction for the chlorine anion, 710.2 mg kg^−1^, against 638.5 mg kg^−1^ from bromine. Therefore [C_8_MIm] Cl was selected for further optimization through design of experiments (DoE). Results were counterposed with those achieved in a previous optimization study using ethanol/water as extractive solvent. The authors found that the results for the ethanolic extract are in the same order of magnitude of [C_8_MIm] Cl, which could be sufficient for justifying its use in this process. Besides that, the environmental fate of ethanol/water mixtures is considerably milder compared to [C_8_MIm] Cl, and its higher extraction efficiency makes it preferable than the extraction with choline acetate. After the optimization, the two extracts obtained with [C_8_MIm] Cl and ethanol were investigated for their algae growth inhibition activity to explore their potential as e.g., antifouling agents.

Calvo-Flores and Martin-Martinez have provided a comprehensive tutorial review on the state-of-the-art of biorefineries. The concept, arose during the late 1990s, deal with the critical solution to co-produce materials, sustainable fuels, and platform chemicals from a great diversity of non-edible biomass feedstocks. The authors pointed out the definition of biomass like any organic matter, derived from living, or recently living animals or plants such as crops (dedicated production), as well as the waste derived from them, or other residues from municipal wastes, wastewater treatment, and feedstock. According to the technology used in the biomass conversion, biorefineries implement two main types of platforms, which can be used in a single way or combined: thermochemical platforms and biotechnological platforms (detailed later). Biomasses can be processed through the one platform or different platforms at the same time. According to this, biorefineries are classified into phase I (use a single feedstock, e.g., biodiesel biorefineries), phase II (carry out a set of processes to produce different products from a single feedstock material) and phase III (produce multiple types of products from multiple feedstock materials through a diverse processing technology). Four different subgroups may be identified for the latter: a) whole-crop biorefinery, in which raw crops such as wheat or corn are used as a unique feedstock material to produce value-added products such as chemical building blocks, pharmaceutical products, textiles, plastics, lubricants, and biofuels; b) green biorefinery, where natural-wet biomass such as cereals or grass are processed and transformed into marketable chemicals and fuels; c) lignocellulosic biorefinery, value-added products (e.g., bio-oil, biochar, or other bio-based chemicals) are produce from lignocellulosic materials; and d) two-platform concept biorefinery, where both thermochemical and biochemical conversions take place in an integrated design to produce valuable products and fuels. Then the main pre-processing and processing technologies generally used in biorefinery are described and classified into four clusters. The (i) mechanical–e.g., pressing, fractionation, and size reduction- and (ii) chemical–e.g., acid hydrolysis, oxidations, and esterification-processes usually used as pre-processing techniques to breakdown biomass before getting into a (iii) thermochemical or (iv) biochemical process. In thermochemical platforms (iii), biomass undergoes high temperature processing, many times combined with high pressures, and with or without the presence of solvents and catalysts. These platforms are mostly focused on the production of biofuels, although there are some cases in which chemicals are also produced at high temperature in an oxygenic or anoxygenic environment. The main technologies applied in thermochemical platforms are combustion, carbonization, gasification, pyrolysis and hydrothermal processing. Biotechnological platforms (iv) are based on the enzymatic conversion of biomass, that is, fermentations. The conversion process occurs under milder conditions than in the thermochemical platforms and they can be performed under aerobic or anaerobic conditions, or under combinations of them. These platforms are mostly focused on the preparation of high value chemicals. Although the differences between different groups of biotechnological platforms in biorefineries are sometimes subtle, they can be generally grouped in a) syngas platform, b) biogas platform, c) C5/C6 carbohydrates platform, d) lignin platform and e) plant-based oil. In the latter case two main platform technologies exist, the algae-based and the press juice platform. Finally, the authors provide a comprehensive list of the main biorefinery products.

Pappalardo et al. have reported the preparation of different sulfate zirconia for chitin and chitosan depolymerization. Herein, chitin and chitosan are abundant unique sources of biologically-fixed nitrogen mainly derived from residues of the fishery productive chain. Their high potential as nitrogen-based highly added-value platform molecules is still largely unexploited and a catalytic way for their valorization would be strongly desirable within a biorefinery concept. The authors report their results obtained with a series of heterogeneous catalysts in the depolymerization of chitosan and chitin to acetylglucosamine. Copper catalysts supported on SiO_2_, SiO_2_–Al_2_O_3_, SiO_2_-ZrO_2_, ZrO_2_ and the corresponding bare oxides/mixed oxides were tested, together with a sulfated zirconia system (ZrO_2_-SO_3_H) that revealed to be extremely selective towards glucosamine, both for chitosan and chitin, thus giving pretty high yields with respect to the values reported so far (44% and 21%, respectively). The use of a heterogeneous catalyst alone, without the need of any additives or the combination with a mineral acid, makes these results remarkable. This protocol is the first example relying on the use of a heterogeneous catalyst alone, active without the addition of any additives or mineral acids. This represents a step ahead, compared to homogeneous-catalysed processes that require massive neutralisation and purification steps, or the enzymatic ones that suffer for high costs and long reaction times.

Finally, Zhang et al. discussed on the important topic of glycerol biorefinery in a very exhaustive review. They investigated literature about combined dehydrogenation of glycerol with catalytic transfer hydrogenation of H_2_ acceptors to chemicals.

Catalytic transformation of low-cost glycerol to value-added lactic acid (LA) is considered as one of the most promising technologies for the upgradation of glycerol into renewable products. Currently, research studies reveal that anaerobic transformation of glycerol to LA could also obtain green H_2_ with the same yield of LA. However, the combined value-added utilization of released H_2_ with high selectivity of LA during glycerol conversion under mild conditions still remains a grand challenge. In this perspective, for the first time, the authors conducted a comprehensive and critical discussion on current strategies for combined one-pot/tandem dehydrogenation of glycerol to LA with catalytic transfer hydrogenation of H_2_ acceptors (such as CO_2_) to other chemicals. The aim of this overview was to provide a general guidance on the atomic economic reaction pathway for upgrading low-cost glycerol and CO_2_ to LA as well as other chemicals. In this review, plausible reaction pathways and mechanisms for catalytic upgradation of glycerol into LA under both aerobic and anaerobic conditions, one-pot/tandem dehydrogenation and catalytic transfer hydrogenation between glycerol and H_2_ acceptors have been critically reviewed with the aim to provide insights into future development of the reaction pathways of atomic economy during process development in catalytic upgradation of unconventional resources to value-added fuels and chemicals. A variety of different H_2_ acceptors have been proposed with remarkable performance for transfer hydrogenation with released H_2_ from dehydrogenation of glycerol. Plausible reaction pathways and mechanisms have been well documented in the current work.

